# Old Age and Frailty in Deceased Organ Transplantation and Allocation–A Plea for Geriatric Assessment and Prehabilitation

**DOI:** 10.3389/ti.2023.11296

**Published:** 2023-07-05

**Authors:** Arved Weimann, Marlies Ahlert, Daniel Seehofer, Tania Zieschang, Mark Schweda

**Affiliations:** ^1^ Department of General, Visceral and Oncological Surgery, St. George Hospital, Leipzig, Germany; ^2^ Department of Economics, Martin-Luther-University of Halle, Halle, Germany; ^3^ Department of Visceral, Transplantation, Thoracic and Vascular Surgery, University Hospital Leipzig, Leipzig, Germany; ^4^ Division of Geriatric Medicine, Department of Health Services Research, School of Medicine and Health Sciences, University of Oldenburg, Oldenburg, Germany; ^5^ Division of Medical Ethics, Department of Health Services Research, School of Medicine and Health Sciences, University of Oldenburg, Oldenburg, Germany

**Keywords:** old age, frailty, organ allocation, organ transplantation, prehabilitation

## Abstract

Due to demographic ageing and medical progress, the number and proportion of older organ donors and recipients is increasing. At the same time, the medical and ethical significance of ageing and old age for organ transplantation needs clarification. Advanced age is associated with the frailty syndrome that has a negative impact on the success of organ transplantation. However, there is emerging evidence that frailty can be modified by suitable prehabilitation measures. Against this backdrop, we argue that decision making about access to the transplant waiting list and the allocation of donor organs should integrate geriatric expertise in order to assess and manage frailty and impairments in functional capacity. Prehabilitation should be implemented as a new strategy for pre-operative conditioning of older risk patients’ functional capacity. From an ethical point of view, advanced chronological age *per se* should not preclude the indication for organ transplantation and the allocation of donor organs.

## Introduction

In the Eurotransplant region, the trend of increasing age of both donors and recipients of deceased donor organs is evident. Between 2012 and 2021, the share of deceased donors older than 65 years rose from 23.3% to 27.8%. A similar tendency can be shown for recipients. In 2012, 3.88% of the recipients of lungs were older than 65. In 2021, this share amounted to 12.64%. In the same time interval, there was an increase of the respective shares of older recipients of livers from 13.45% to 19.39%, of hearts from 6.68% to 9.29%, and of kidneys from 27.4% to 28.98% [1].

These increases in donor and recipient age raise new questions in public and policy debates on organ donation in the context of old age [2, 3]. Thus, the growing number of older potential organ recipients intensifies concerns about “organ scarcity” and fuels controversies about the efficient use and just distribution of available donor organs between age groups [4, 5]. At the same time, however, older people are discovered as a largely untapped source of donor organs and play an important role in new strategies for a more efficient and fair utilization of available organs. For example, they are targeted as a separate subgroup of kidney donors and recipients in “old for old” programs like the Eurotransplant Senior Program (ESP) [2].

In these and similar debates, assumptions about the medical chances and risks of organ transplantation at advanced age play a crucial role. The prospective medical success of the procedure is a criterion for its medical indication and ethical beneficence at an advanced age. It also factors into the discussion and regulation of the appropriate allocation of donor organs. In this context, advanced chronological age is frequently discussed as a distribution criterion, fostering controversial proposals for age-based rationing of medical resources for the sake of younger age groups and sparking concerns about age discrimination [6, 7].

A central aspect is the functional capacity of older recipients in the context of frailty. The frailty concept describes a syndrome which is associated with ageing and means impairment of functional capacity, physiological reserve, and body resilience. In older people undergoing major surgery, these changes may bear a considerable risk for the development of postoperative complications and prolonged recovery, including limited graft function in the case of organ transplantation. Functional status declines on the waiting list for kidney transplantation and has been shown to be associated with greater mortality and all-cause graft loss [8]. Similar findings have also been reported for liver transplantation [9]. However, recent studies indicate that this risk may be modifiable through adequate preventative measures. Therefore, it may be medically unwarranted and ethically problematic to exclude patients based on chronological age.

Against this backdrop, the contribution discusses the medical assessment and ethical evaluation of the success of organ transplantation in old age. In doing so, we particularly focus on the relevance of frailty for transplant success. We first provide a brief overview of the increasing relevance of old age in organ donation and transplantation, also considering the role of chronological age and frailty in allocation algorithms like the LAS score for lung transplantation. We then highlight the state of research regarding the impact of frailty on transplantation outcomes and review existing evidence that frailty constitutes a modifiable risk factor which can be mitigated by preventative measures. On this basis, we draw conclusions for an adequate treatment of older patients in organ transplantation. These include appropriate score-based risk stratification to achieve transparency for decision making and allocation algorithms of transplant candidates. Before excluding a candidate from transplantation due to age-related functional impairment and frailty, all potential measures of conditioning the patient should be taken into consideration.

## Old Age and Frailty in Organ Transplantation

There is ample evidence that older patients can benefit from organ transplantation. In the US and Europe, a survival advantage for older people (>60 years) vis-a-vis patients on the waiting list who remain on dialysis could be observed [10]. Compared to dialysis, organ transplantation doubles the life expectancy of older people [11]. Survival improves after the first year in patients between 60 and 74 years with a predicted increased life expectancy of 5 years and a 61% reduction in long-term mortality risk [12, 13]. Even in ESP kidney transplantation, the quality of life and the survival rate are significantly better than in patients of the same age who are dialyzed [14].

Nevertheless, older patients pose certain challenges to transplantation medicine. This is due to functional impairment and considerable comorbidity often related to the underlying organ dysfunction. In recent years, frailty has come into consideration as an identifiable preoperative risk factor for the postoperative outcome of organ transplantation [15–20]. The concept describes a syndrome which is associated with ageing and means impairment of functional capacity, physiological reserve, and body resilience. Frailty symptoms are unintended weight loss, exhaustion, weakness, slow gait speed, and low physical activity. They can be summarized in the Fried Frailty Index or other indices that also consider cognitive functioning [21, 22]. While age is the only conventional factor associated with frailty in kidney transplant patients, activities of daily living (ADL), depression scale, education, and health-related quality of life (HRQOL) are independently associated. Poor grip strength, exhaustion, and slowed walking speed are predictors for mortality risk [23]. Moreover, preoperative cognitive function in older people has turned out to be associated with postoperative complication rate and length of hospital stay after major surgery [24].

Frailty is also frequently associated with sarcopenia [25]. The International Working Group on Sarcopenia (IWGS) defines sarcopenia as “age-associated loss of skeletal muscle mass and function.” The primary parameter is reduced muscle strength which leads to impaired physical resilience due to reduced muscle quantity or quality [26, 27]. Primary sarcopenia is age-associated, whereas secondary sarcopenia has other causes, e.g., a systemic disease, increased inflammation, decreased physical activity, and inadequate energy and protein intake [27]. Chronic organ failure as the indication for organ transplantation is frequently associated with sarcopenia and frailty [28, 29]. Sarcopenia has been shown to be an independent predictive factor of postoperative complications after liver transplantation for primary liver tumors [30], as well as for major morbidity and mortality after lung and heart transplantation [25, 31]. There is controversial data regarding the correlation of sarcopenia and long-term survival after liver transplantation [30, 32].

A high number of hospital admissions has been observed for kidney transplant candidates during the first year on the waiting list, which is a risk factor for waiting list mortality and lower graft and recipient survival [33]. Most of the symptoms are common across different types of organ failure. A systematic review of frailty in lung transplantation showed a prevalence of frailty of 0%–58% [34]. In kidney transplant recipients, prevalence of frailty is about 11% and has been shown to be associated with dialysis duration [35]. Frailty is a predictor of surgical complications after kidney transplantation [20, 36]. In patients undergoing lung transplantation, frailty was associated with decreased survival and an increased risk of early mortality in a systematic review [34]. The syndrome may be also associated with postoperative delirium and medium-term cognitive decline after transplantation [8, 37]. Furthermore, discharge frailty is also associated with a risk for unplanned rehospitalization [38]. Importantly, a prospective study in kidney transplant recipients showed that pretransplant frailty may improve after an initial decline within 3 months after surgery [39].

Frailty thus constitutes a highly relevant aspect in the consideration of organ transplantation in older adults. Geriatric medicine has developed authoritative expertise and instruments to detect and assess frailty. Comprehensive geriatric assessment (CGA) is a multidimensional, multidisciplinary process which identifies medical, social, and functional needs, and the development of an integrated/coordinated care plan to meet those needs [40]. The instruments used in CGA allow for the identification but also quantification of risk factors, functional capacities and impairments, as well as needs and strengths/resilience of an individual person in his or her environmental setting and goes beyond the determination of frailty status. Importantly, with the help of CGA, modifiable risk factors can be identified and consecutively targeted by interventions such as exercise, nutrition, adaption of medication, or prehabilitation. Components of CGA include assessments regarding medical/physical, psychological/psychiatric (cognition, emotion), functionality, mobility and falls, nutrition, socio-economic aspects through which goal setting, care planning, treatment/rehabilitation as well as discharge planning are tailored for the individual patient [40].

In the context of organ transplantation, a study on incorporating geriatrics and geriatric assessments into kidney transplant evaluation showed that this was feasible and that components of the geriatric assessment, specifically walking speed, falls, dependencies in the Instrumental Activities of Daily Living (IADL) and Activities of Daily Living (ADL), were significantly associated with patients’ transplant rate, waiting list placement or removal, and mortality [41]. Another study on using CGA for decision making concerning kidney transplant revealed that geriatricians’ recommendations for kidney transplant was influenced by impairments in IADL, physical function, and frailty [42].

## Old Age and Frailty in Organ Allocation

Old age and frailty also play an important role in the allocation of donor organs. Special transplantation programs for older people have been existing for over 20 years. In the United States, the allocation of kidneys divides donors into standard kidney donor profile index patients (KDPI) and high kidney donor profile index patients (high KDPI). High KDPI kidneys derive from donors older than 60 years and donors 50–59 years with co-morbidities. Participation in this allocation scheme is voluntary and one can choose to be listed for the KDPI kidneys (opt in). The vast majority of patients on the KDPI waiting list are older candidates. For older people, an advantage of this system is that it uses an age-matching formula whereby recipients are entitled to kidneys from donors who are no more than 15 years younger or older [43].

In the Eurotransplant region, organ-specific allocation rules differ with respect to the incorporation of age or functionality-related variables. Age is an explicit criterion in the allocation of kidneys and lungs, whereas variables measuring functionality are only explicitly relevant in lung allocation. The Eurotransplant Senior Program (ESP) was established in 1999 as a special program for kidney transplantation from older donors to older recipients [44]. The program allocates organs between donors and recipients who are 65 years and older [11, 45]. Since 2001, the ESP has become part of the Eurotransplant Kidney Allocation System (ETKAS) [46]. Germany, the Netherlands, and Belgium are the most important contributors [47]. Using regional allocation based on waiting time and blood group only, regardless of HLA match, a short cold ischemic time (CIT) and thus a good primary organ function can be achieved [48]. ESP leads to significantly reduced waiting times and enhances the chance for older patients to receive a renal graft [11].

An example for the role of age and its relation to functionality in organ allocation is the lung allocation score (LAS). The LAS has constituted the basis for the priority rule of lung allocation in Germany since May 2005. The higher a patient’s LAS, the higher his or her priority to receive a donor lung. The score is constructed based on empirical data from the United States. It consists of estimates for urgency and expected survival after transplantation at the time an organ is offered. For each patient on the lung waiting list, specific data characterizing the patient and their health status are needed.

The first element of a patient’s LAS is an estimate of urgency based on the estimated probabilities to survive from day to day without a transplant during the next year. The second element consists of estimates for day-to-day survival within the year after transplantation. For these estimates, several diagnostic data are used, among them the variables age at the time of offer (depending on the type of diagnosis), functional status (distinguishing between no assistance, some assistance and total assistance) and 6-min walking distance (more than 150 feet or not). Ceteris paribus, the older the patient, the lower their functional status or walking distance, the shorter the expected survival without a transplant, i.e., the higher the estimated urgency. Ceteris paribus, the higher the age at transplantation, the lower the expected survival time after transplantation.

The estimate for transplantation success in the LAS is the difference between the expected survival time with transplant and without transplant. Since both are influenced negatively by age, it is not possible to make a general statement on the dependency of the success measure on age. The LAS itself is constructed as a difference of the measure for success and the expected survival time without a transplant. The higher the urgency, the higher the LAS, the higher the estimated success the higher the LAS. Although a general statement on the ceteris paribus dependency of the LAS on age is not possible, comparing fictional examples shows, e.g., that patients of higher age can achieve a relatively high LAS in case they need no assistance or some assistance. Comparable approaches integrating functional capacity measures are missing for other types of organ transplantation.

From a geriatric point of view, chronological age *per se* should not have too much impact on the allocation score. Instead, functional capacity should be given greater weight. Although walking speed is a major predictor of functional decline and mortality in older people [49], the internationally widely used Short Physical Performance Battery (SPPB) [50] additionally measures balance control and lower limb strength (five chair rise). The SPPB has been used to assess physical function in a study on pre-transplant physical function and outcomes after kidney transplant [51]. It was shown to be independently associated with length of hospital stay regardless of age [52, 53] and is also a common measure used in lung transplantation [54]. Generally, in geriatrics, assessment of cognition is important and cognitive impairment is also considered part of a frailty phenotype. However, in a study on frailty measures in patients listed for lung transplantation, cognitive function and depression variables did not strengthen the association with lung transplant waiting list mortality compared with the physical frailty measure [55]. Further research is needed to assess the influence of cognitive impairment on transplantation outcomes.

## Prehabilitation and Transplant Success

Age and functionality represent important factors in the assessment of transplant success. Therefore, they also play a crucial role regarding access to and allocation of donor organs. Thus, it has been shown in a prospective multicenter study that frailty is associated with a lower chance to be listed for kidney transplantation [15]. However, there is increasing evidence that impaired functionality due to frailty may be a modifiable risk factor in older patients. For example, heart-failure associated frailty may be reversible [56]. In a study on lung transplant patients, pre-transplant SPPB increased following pre-habilitation [57].

**FIGURE 1:**
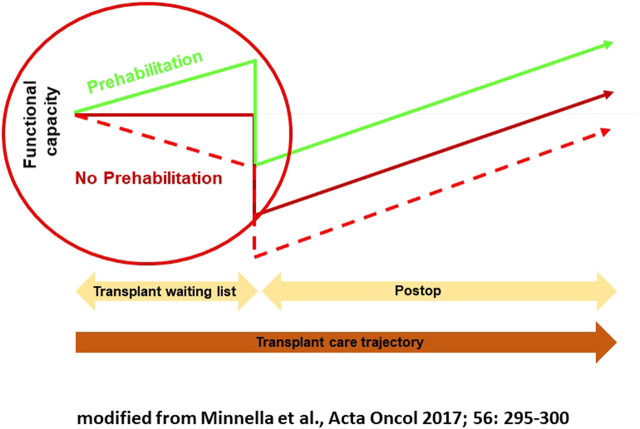
Multimodal prehabilitation.

Preoperative conditioning can improve physical function and nutritional status in high-risk patients before major abdominal surgery and may reduce the rate of complications [58, 59]. So-called trimodal prehabilitation consists mainly of physiotherapy and nutrition therapy as well as psychological intervention. New data suggest that long-term preoperative conditioning performed in appropriate risk patients not only improves physical functions and nutritional status *per se* but can also have positive effects on the postoperative course [60]. The concept developed by a Canadian group with anesthetist Franco Carli is to improve the functional status before the operation in order to attenuate the postsurgical decline, to diminish the risk for a complicated course, and to treat the patient according to an enhanced recovery after surgery (ERAS) protocol (Figure 1) [58, 59].

The exact effects of prehabilitation on the postoperative systemic inflammatory response have not been elucidated, yet. When comparing prehabilitation in major surgery with conventional rehabilitation alone, the additional prehabilitation appears to be more effective. A period of four to 6 weeks has been proven efficient in patients undergoing major surgery for cancer. A recent meta-analysis of 22 randomized studies carried out between 1991 and August 2020 showed a significant improvement in functional capacity for patients undergoing major cancer surgery, measured in 6-min walking distance, as well as a significantly shorter hospital stay [60]. In other meta-analyses, a decrease of complication rate with special regard to pulmonary morbidity has been observed [61, 62]. In a recent multicentric randomized clinical study in patients undergoing surgery for colorectal cancer, a 4-week in-hospital supervised multimodal prehabilitation was investigated. 251 patients were analyzed regarding intention-to-treat. The number of severe complications was significantly lower in the treatment group compared to standard care, and prehabilitation patients had significantly fewer medical complications [63].

Similar experience is currently beginning to emerge for the field of organ transplantation. Thus, prehabilitation has been shown feasible prior to kidney transplantation in a Johns Hopkins pilot study of 24 patients [64]. Remote coaching of home exercise has also been proven to be feasible and effective in patients on the waiting list for kidney transplantation [65]. Furthermore, the feasibility of a 12-week home-based prehabilitation was demonstrated in 18 candidates for liver transplantation with an improvement of aerobic and functional capacity, as well as parameters of quality of life. The program included average daily step targets and twice-weekly resistance exercise [66]. Eventually, prehabilitation has also been shown to be effective for improving quality of life and mood status, and reducing dyspnea in patients waiting for lung transplant [67]. Nevertheless, evidence is still limited, especially with regard to duration, modalities and intensity of the program. More systematic research with well-powered randomized trials is needed. Recently, a protocol for a comparative study of frailty in patients on the kidney transplant list regarding the composite of time to death or permanent waiting list withdrawal was published in Canada [68]. Secondary outcomes will include number of hospitalizations and length of stay, and in a subset, changes in frailty severity over time, changes in quality of life, and the probability of being accepted to the waiting list.

## Conclusion and Outlook

Old age and frailty play a crucial yet complex role in organ transplantation and allocation. In light of geriatric research, a general equation of advanced chronological age and frailty appears unacceptable. Moreover, there is increasing evidence that frailty constitutes a modifiable risk factor that can be mitigated by suitable prehabilitative measures.

This has important implications for transplantation medicine. First, general chronological age limits for organ transplantation and allocation appear problematic. The functional status and thus the chances and risks of organ transplantation for older patients need to be assessed on an individual basis. When it comes to organ allocation, complex, multifactorial score systems incorporating geriatric scores provide a more accurate, differentiated, and transparent account than general age limits. In both contexts, geriatric medicine can offer suitable professional expertise and validated tools, such as the widely used SPPB that has already been applied in several studies on patients waiting for lung or kidney transplant.

At the same time, the potentials of prehabilitation to mitigate the risks and increase the success rates of organ transplantation for older recipients need further scientific examination and evidence-based practical guidelines. To this end, more systematic data collection and large-scale clinical studies are needed to investigate the effects of prehabilitation and evaluate and compare the outcomes of different prehabilitation measures, especially in the context of organ transplantation for older people, with a focus on health-related quality of life [69]. On this basis, specific guidelines for clinical practice could be formulated.

There are currently no clear recommendations for the organization and implementation of prehabilitation programs. Programs vary widely in terms of duration, content, and frequency of individual measures. For transplant candidates, home- or community-based programs will be most favorable. The special challenge in transplantation patients is the unpredictable time of surgery, and the motivation of the patient for self-managing responsibility. At the same time, motivation and cooperation of the patient in prehabilitation may be considered a predictor of long-term adherence as a basic requirement for transplant success. There are first approaches to prepare and support older transplant recipients for self-management before transplantation, to clarify expectations regarding posttransplant outcome, and to provide support in case of prolonged recovery [70].

Overall, the realization of these recommendations requires a systematic inclusion of geriatric expertise in the relevant studies, organizations, and clinical procedures in the field of transplantation medicine. Geriatric professionals and assessment instruments for frailty like the SPPB should be included on a regular basis in the evaluation of older potential transplant recipients. More research and practical experience is needed regarding the successful involvement of geriatricians in the process of waiting list placements. In addition, state of the art geriatric research should inform the formulation of adequate allocation scores and algorithms for older patients, as well as the development and implementation of suitable prehabilitation programs. This can help to support a more effective utilization of donor organs and prevent ageist stereotypes as well as fears of discrimination of older people in the context of organ transplantation.

## Data Availability

The original contributions presented in the study are included in the article/supplementary material, further inquiries can be directed to the corresponding author.
